# Advances in holliday junction recognition protein (HJURP): Structure, molecular functions, and roles in cancer

**DOI:** 10.3389/fcell.2023.1106638

**Published:** 2023-03-21

**Authors:** Lin Li, Qiang Yuan, Yue-Ming Chu, Hang-Yu Jiang, Ju-Hua Zhao, Qiang Su, Dan-Qun Huo, Xiao-Fen Zhang

**Affiliations:** ^1^ Key Laboratory for Biorheological Science and Technology of Ministry of Education, Bioengineering College of Chongqing University, Chongqing, China; ^2^ Department of Pharmacy, The Second Clinical Medical College of North Sichuan Medical College, Nanchong, China; ^3^ School of Pharmacy, North Sichuan Medical College, Nanchong, China; ^4^ Institute of Tissue Engineering and Stem Cells, The Second Clinical Medical College of North Sichuan Medical College, Nanchong, China; ^5^ Nanchong Key Laboratory of Individualized Drug Therapy, Nanchong, China

**Keywords:** HJURP, CENP-A, centromere, cancer, prognosis

## Abstract

Oncogenes are increasingly recognized as important factors in the development and progression of cancer. Holliday Junction Recognition Protein (HJURP) is a highly specialized mitogenic protein that is a chaperone protein of histone H3. The HJURP gene is located on chromosome 2q37.1 and is involved in nucleosome composition in the mitotic region, forming a three-dimensional crystal structure with Centromere Protein A (CENP-A) and the histone 4 complex. HJURP is involved in the recruitment and assembly of centromere and kinetochore and plays a key role in stabilizing the chromosome structure of tumor cells, and its dysfunction may contribute to tumorigenesis. In the available studies HJURP is upregulated in a variety of cancer tissues and cancer cell lines and is involved in tumor proliferation, invasion, metastasis and immune response. In an *in vivo* model, overexpression of HJURP in most cancer cell lines promotes cell proliferation and invasiveness, reduces susceptibility to apoptosis, and promotes tumor growth. In addition, upregulation of HJURP was associated with poorer prognosis in a variety of cancers. These properties suggest that HJURP may be a possible target for the treatment of certain cancers. Various studies targeting HJURP as a prognostic and therapeutic target for cancer are gradually attracting interest and attention. This paper reviews the functional and molecular mechanisms of HJURP in a variety of tumor types with the aim of providing new targets for future cancer therapy.

## 1 Introduction

According to current global disease statistics, cancer has surpassed all other diseases in terms of morbidity and mortality (Sung et al., 2021). Although the mechanisms of tumorigenesis and cancer progression have been extensively studied, further research is essential to advance treatment options and improve treatment outcomes. Back in 2007, a novel gene, initially annotated as fetal liver-expressing gene 1 (hFLEG1) and later termed Holliday junction recognition protein (HJURP), was found to show a fold change expression greater than five in non-small cell lung cancer compared to normal tissue (Kato et al., 2007). Research showed also that the serine-threonine kinase Akt/PKB can phosphorylate HJURP *in vitro*, and the acronym FAKTS (14-3-3-associated AKT substrate) was coined to refer to the phosphorylated HJURP form (Luhn et al., 2007). The HJURP gene is located in the chromosomal region 2q37.1 and is approximately 17.73 kb in length with nine exons. The full-length cDNA sequence of HJURP is 2,529 nucleotides long and the open reading frame consists of 2,244 nucleotides encoding 748 amino acids (Sanchez-Pulido et al., 2009; Hu et al., 2011; Zasadzińska et al., 2013). HJURP was identified as a distinct CENP-A histone chaperone protein that mediates the assembly, maintenance, and deposition of CENP-A nucleosomes in mitotic chromatin (Dunleavy et al., 2009; Foltz et al., 2009). HJURP exerts important molecular functions through binding to structural domains of CENP-A to form a multimeric complex that regulates mammalian chromosome stability, promotes chromosome segregation and cell mitosis, and restores DNA double-strand breaks (DSBs) (Dunleavy et al., 2009; Foltz et al., 2009).

Aberrant HJURP expression is common in tumors, including hepatocellular carcinoma (Chen T. et al., 2019), renal cell carcinoma (Zhang et al., 2021), ovarian cancer (Dou et al., 2022), osteosarcoma (Jia et al., 2022), pancreatic cancer (Wang C. J. et al., 2020), and oral cancer (Tsevegjav et al., 2022). Abnormal activation of HJURP may be linked to unrestricted proliferation of tumor cells (Kato et al., 2007), an effect associated with enhanced chromosomal stability and promotion of DNA DSB repair *via* the homologous recombination pathway (Mishra et al., 2011; Vishwakarma and McManus, 2020). Still, despite recent studies indicating that HJURP dysregulation contributes to the progression of a variety of human cancers, little is known about the specific mechanisms.

In this article, we summarize the available data on the structure, localization, and molecular functions of HJURP and review its pathogenic role in human cancers, addressing its potential as a diagnostic, prognostic, and therapeutic cancer marker.

## 2 Structural characteristics and subcellular localization of HJURP

**FIGURE 1:**
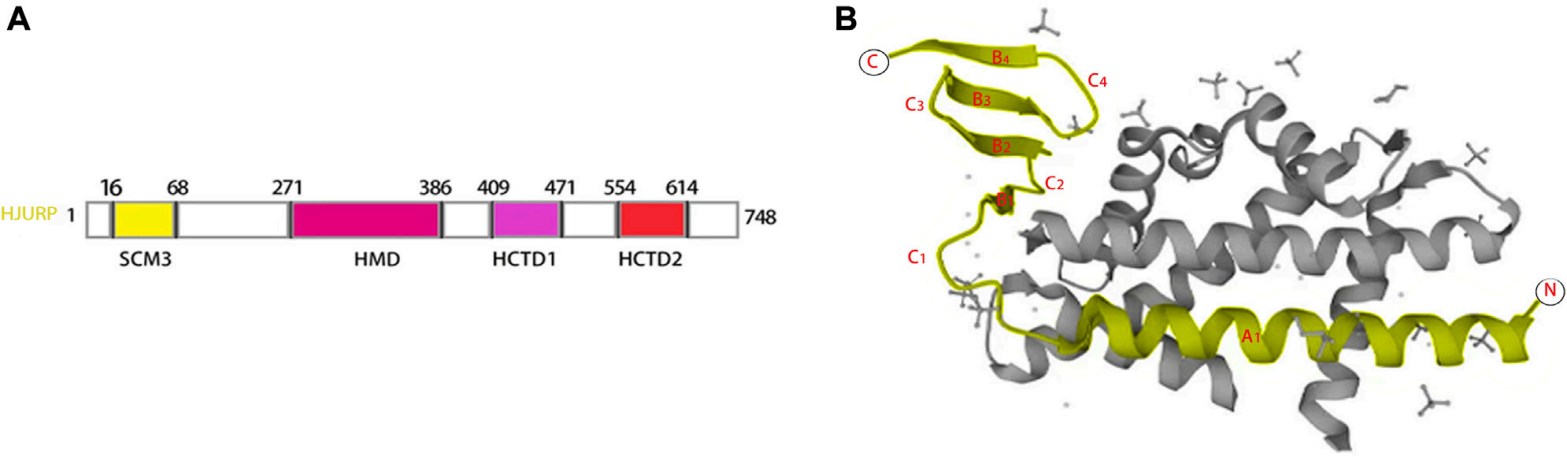
Secondary structure and crystal structure of HJURP. The HJURP gene has four secondary structural domains SCM3, HMD, HCTD1, and HCTD2, respectively **(A)**.The crystal structure of HJURP consists of a long helix (A1) and a triple antiparallel β-sheet connected by a 15-residue ring (C1) **(B)**.

HJURP contains four structural domains suppressor of chromosome missegregation protein 3 (SCM3 16–68), HJURP middomain (HMD 271–386), HJURP C-terminal domain 1 (HCTD1 409–471), and HJURP C-terminal domain 2 (HCTD2 554–614), separated by variable length insert sequences (Sanchez-Pulido et al., 2009; Zasadzińska et al., 2013) (Figure 1A). The N-terminal histone-binding domain of HJURP interacts with CENP-A as a pre-deposited complex with histone H4 (Shuaib et al., 2010; Hu et al., 2011; Bassett et al., 2012). The SCM3 domain, which is a distal homolog of yeast Scm3, is a CENP-A binding site that promotes CENP-A nucleosome formation *in vivo* and *in vitro* (Pidoux et al., 2009; Shuaib et al., 2010; Barnhart et al., 2011; Muller et al., 2014). The interaction of HJURP with CENP-A necessitates the presence of a TLTY box (Shuaib et al., 2010), and loading of CENP-A at centromeres requires direct interaction of HJURP with DNA *via* a conserved DNA-binding region in the HMD domain (Muller et al., 2014). HJURP is phosphorylated at the HCTD1 during the majority of the cell cycle and dephosphorylated at the end of mitosis, coinciding with cyclin-dependent kinase (CDK1/CDK2) downregulation. This dephosphorylation causes HJURP to be recruited to centromeres during telophase (Zasadzińska et al., 2013; Muller et al., 2014). Ser412, Ser448, and Ser473 are key CDK targets during the M/G1 transition phase (Muller et al., 2014). The HCTD2 domain mediates HJURP dimerization, an essential event for centromeric CENP-A loading (Zasadzińska et al., 2013). This is achieved by binding of an HJURP homodimer to a CENP-A-histone H4 heterodimer; upon dimerization, HJURP may bring two new CENP-A-H4 heterodimers to centromeres to form an octameric nucleosome (Zasadzińska et al., 2013). The CENP-A tetramer, consisting of CenH3, H4, H2A and H2B, is also known as the “hemispheric structure” (Dalal et al., 2007; Lavelle and Prunell, 2007). In humans, complexation of HJURP with CENP-A/H4 heterodimers is mediated by a conserved HJURP short N-terminal domain termed CENP-A-binding domain (CBD) (Hu et al., 2011) (Figure 1B). The CBD domain comprises amino acids 14–74 and consists of a lengthy helix (A1) and a three-stranded anti-parallel β-sheet joined by a loop of 15 residues (C1) (Hu et al., 2011) (Figure 1B). Polar interactions are established between Glu96 of CENP-A and Arg32, Lys39, and Tyr40 of HJURP, between Glu107 of CENP-A and Arg28 of HJURP, and between His104 and Asp108 of CENP-A and Ser25 of HJURP (Hu et al., 2011). Hydrophobic bonds mostly mediate side chain interactions between HJURP and CENP-A, which involve amino acids from C1 and the β-sheet domain of HJURP. In turn, HJURP residues within the A1-C1 linkage mediate direct contact with histone H4 by a combination of hydrophobic and van der Waals interactions (Hu et al., 2011). HJURP localizes to centromeres at the end of CENP-A nucleosome assembly and during early G1 phase, and to the nucleolus during the S phase (Dunleavy et al., 2009). Interestingly, immunohistochemical assays indicated that HJURP localized mainly in the cytoplasm in both prostate cancer (Chen Y. F. et al., 2019; Lai et al., 2021) and hepatocellular carcinoma (Chen et al., 2018; Chen T. et al., 2019) cells. Meanwhile, HJURP expression was detected in both the cytoplasm and nucleus of gliomas (De Tayrac et al., 2013).

## 3 Molecular functions of HJURP

### 3.1 Centromere formation

Centromeres play an important role in cell division by coordinating precise segregation of chromosomes into progeny cells (Vagnarelli et al., 2008). Numerous studies have shown that abnormal centromere function can lead to abnormal chromosome segregation, thereby promoting tumor development (Daniela et al., 2001; Goncalves Dos Santos Silva et al., 2008; Thompson and Compton, 2008; Turajlic and Swanton, 2016; Jamal-Hanjani et al., 2017; Bakhoum et al., 2018). Centromere segregation is governed by centromeric proteins such as CENP-A, a variant of histone H3 (Gascoigne et al., 2011). CENP-A maintains centromere localization and controls centromere formation, which is essential for high-fidelity chromosome segregation (Kim et al., 2016). Sequence analysis showed that CENP-A has a total length of 8,534 bp and is located at 2p24-p21 (Li and Zhu, 2006). CENP-A is involved in regulating chromosome structure during mitosis and meiosis, recruiting transcription factors, and regulating gene transcription and DNA damage repair through phosphorylation events (McKinley and Cheeseman, 2016). Recruitment of CENP-A at centromeres requires the help of centromere-specific proteins such as CENP-C, Mis18, and HJURP, among others (Hayashi et al., 2004; Dunleavy et al., 2009; Foltz et al., 2009; Barnhart et al., 2011; Moree et al., 2011; Falk et al., 2015). The localization of HJURP at centromeres is cell-cycle regulated, and CENP-A expression is reduced after inhibition of HJURP (Zasadzińska et al., 2013). Upon centromeric localization, formation of HJURP protein dimers through its carboxy-terminal domain is a critical factor in recruiting CENP-A protein to centromeres (Zasadzińska et al., 2013). In a study using chicken DT40 cells, conditional HJURP knockout led to growth arrest and cells began to die after by 48 h of culture. Immunofluorescence further showed that CENP-A expression at centromeres was reduced in HJURP-deficient cells, a phenomenon accompanied by chromosomal disturbances, including non-disjunction and acentric formation (Perpelescu et al., 2015).

Two homologous structures, Mis18α and Mis18β, exist for the Mis18 protein. The M18BP1 protein is homologous to the *C. elegans* centromeric protein KNL2 and can bind Mis18α/β to form a complex to recruit CENP-A (Perpelescu et al., 2015). It has been found that whereas the carboxy terminus of HJURP is the binding site for CENP-A, the middle region of HJURP is critical for the function of the M18BP1/KNL2 complex (French et al., 2017). The Mis18 protein complex acts also as a receptor for HJURP on centromeres. The middle region (255–500 amino acids) of the HJURP protein is involved in centromere stabilization, and experiments on artificial centromeres revealed that HJURP mutant proteins of different lengths can bind to M18BP1/KNL2 and cause centromere expansion in chromosomes (Pan et al., 2019). Thus, HJURP binds CENP-A at the carboxy terminus, while its middle region can bind Mis18 protein and promote centromere segregation (Wang et al., 2014). Mis18α/β is subject to regulation by kinases such as CDK and polo-like kinase 1 (Plk1). CDKs remain highly active during mitosis and their activity begins to decrease toward the end of this process (McKinley and Cheeseman, 2014). Phosphorylation of CDK negatively regulates centromere localization and Mis18 protein assembly. Plk1 plays the opposite role, positively regulating Mis18 protein assembly and centromere localization, and promoting cell mitosis, upon phosphorylation.

Using the yeast two-hybrid system, Schittenhelm et al. showed that CAL1, *Drosophila’s* HJURP counterpart, directly binds the carboxy terminus of Centromere protein C (CENP-C) (Schittenhelm et al., 2010). This evidence suggested that HJURP can bind CENP-C. Centromere proteins, including CENP-A, Centromere protein B (CENP-B), and CENP-C, is the main component of kinetochore protein. CenH3^CENP−A^ contains an HJURP-binding domain (CATD) and a CENP-C-binding domain (CAC). HJURP binds CATD, and LacI (Lac repressor) fusion of HJURP was shown to drive stable recruitment of CENP-A to a LacO (Lac operon) array at a non-centromeric locus (Tachiwana et al., 2015). In turn, Tachiwana et al. (2015) demonstrated that CenH3^CENP−A^ contributes to the accumulation of CENP-C and Centromere protein T (CENP-T), two proteins necessary for the formation of functional kinetochores, through its CATD and CAC domains (Tachiwana et al., 2015).

In summary, HJURP recruits CENP-A and CENP-C onto centromeres and is essential for maintenance of centromere function and regulation of chromosome segregation. Therefore, abnormal HJURP expression is intimately associated with cell division defects.

### 3.2 Repair of DNA double-strand lesions

Among many types of DNA damage, DSBs are considered the most severe because an unrepaired DSB is sufficient to trigger permanent growth arrest and cell death (Sandell and Zakian, 1993). DSBs mainly activate two repair pathways, namely, homologous recombination (HR) and non-homologous end-joining (NHEJ) (Symington, 2002; Hartlerode and Scully, 2009). The HR pathway is indispensable for protecting genome stability; it is critical both for repairing DNA lesions in mitosis and for chromosomal pairing and exchange during meiosis, thereby ensuring correct segregation of chromosomes during the first meiosis (Nguyen et al., 2020). The ataxia telangiectasia mutated (ATM) kinase belongs to the PI3K family and importantly maintains chromosomal integrity and genomic stability (Nam and Cortez, 2011). ATM is a master regulator of DNA damage, controlling and coordinating DNA replication origins, replication stability, cell cycle checkpoint control, and DNA repair. DSBs recruit and activate ATM kinases, which phosphorylate a series of proteins involved in chromosome and DNA damage repair, directing them to the site of DNA damage primed for HR repair (Smith et al., 2016). Kato et al. studied lung cancer and found that after treatment of A549 cells with λ phosphatase, HJURP showed the synthesized 29 phosphorylated form. Moreover, no expression of HJURP protein was found in cell lines lacking ATM. The same results were obtained using siRNA to interfere with ATM expression (Kato et al., 2007). However, the transcription of HJURP was not affected by ATM. The authors thus suggested that HJURP is a downstream target of the ATM signaling pathway and may be also involved in the repair of DSBs. To prove HJURP involvement in ATM-induced DSB repair, DNA damaging agents such as γ-irradiation, hydroxyurea, and cisplatin were applied to A549 and ATM-deficient cells. Whereas a time- and dose-dependent increase in the expression and nuclear accumulation of HJURP was observed in ATM-competent A549 cells, regardless of treatment HJURP protein expression was abrogated in ATM knockdown cells. It was then inferred that HJURP protects damaged DNA and prevents it from binding sensors of DNA damage, e.g., through failure to activate the ATM kinase, hence inhibiting the DNA damage response (Kato et al., 2007). Until now, only one article has reported the involvement of HJURP in DNA repair, and we expect this aspect to be supplemented in future studies to further confirm its role in DNA repair.

## 4 Roles of HJURP in tumors

**FIGURE 2:**
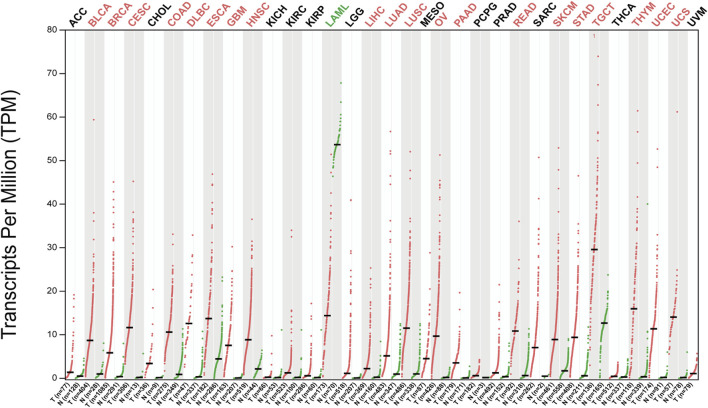
Expression patterns of HJURP in pan-cancer. The expression level of HJURP of difffferent tumor types in the GEPIA database (|Log2Fold Change| > 1 and p < 0.01). Red indicates HJURP high expression in tumor tissues and green indicates HJURP high expression in normal tissues.

**FIGURE 3:**
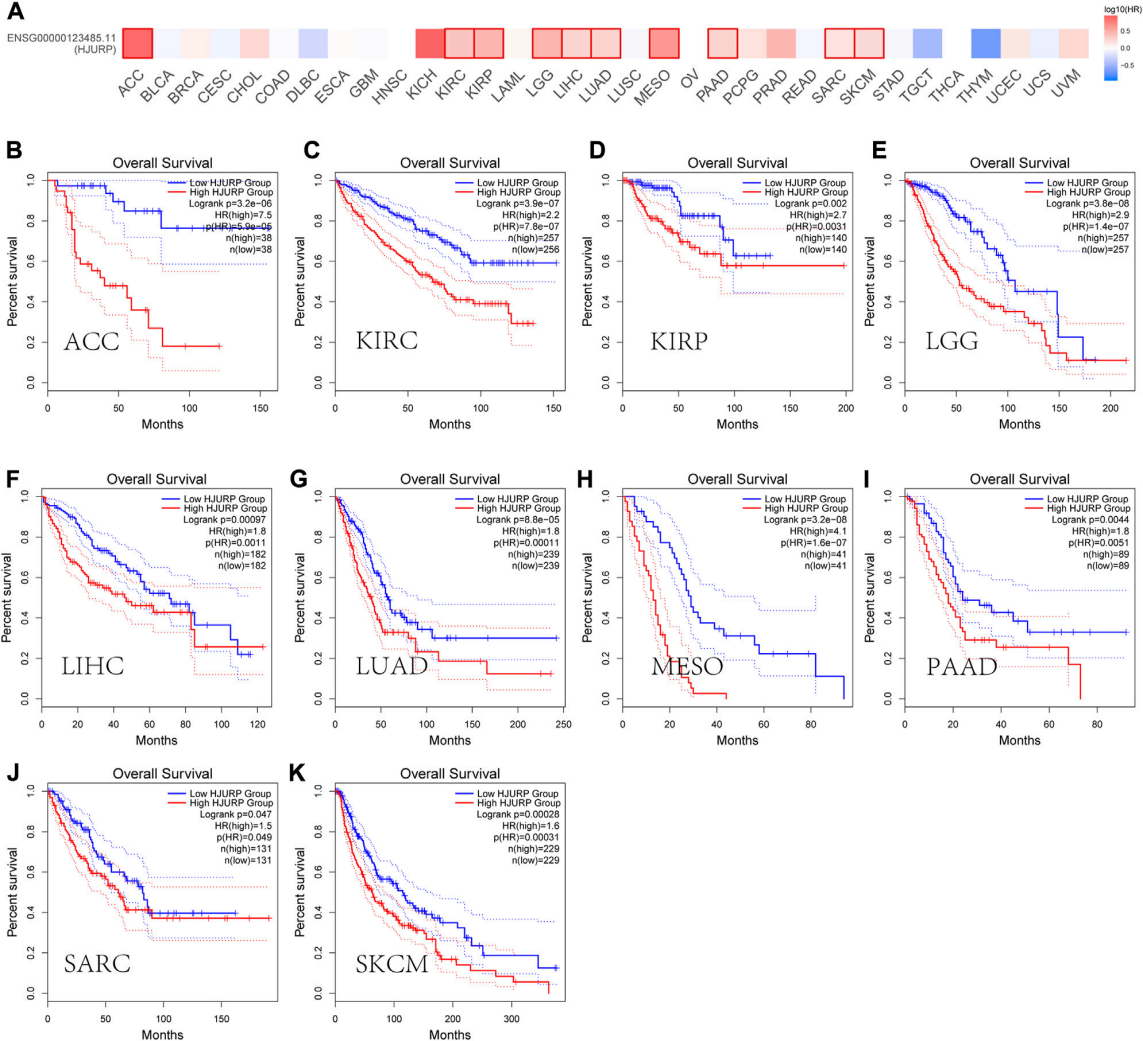
Prognostic roles of HJURP in pan-cancer. **(A)** Prognostic pattern of HJURP in pan-cancer were analyzed using GEPIA database. **(B-K)**The GEPIA database was used to assess the correlation of HJURP with OS of different tumor types.

GEPIA2 (http://gepia2.cancer-pku.cn/) database analysis showed that HJURP expression was higher in tumors tissues than in tumor-adjacent tissue (Figure 2). In addition, most of the tumors with high HJURP expression have poor prognosis (Figure 3). Chromosomal instability is an important feature of human tumors, mainly manifested by frequent loss or gain of chromatin during cell division (Bakhoum et al., 2018). One of the major causes of genetic instability is the failure of chromosomes to segregate correctly in the presence of abnormalities in the expression and function of mitogenic proteins (Hou et al., 2021). Therefore, researchers have extended the study of centromeric protein family to the growth, proliferation, invasion and metastasis of malignant tumor cells (Mahlke and Nechemia-Arbely, 2020). HJURP protein recruits CENP-A and CENP-C to localize to mitoses, maintain mitotic function, and regulate chromosome segregation (Foltz et al., 2009; Moree et al., 2011). The HJURP gene plays a role in promoting cell proliferation, which is closely related to tumorigenesis and may be involved in tumor development (Chen et al., 2018; Lai et al., 2021). Several studies have reported that HJURP acted as an oncogene affecting chromosome segregation and controlling accelerated division of tumor cells (Muller and Almouzni, 2014; Muller et al., 2014). Therefore, increased HJURP expression may be an excellent potential marker of tumorigenesis and disease progression.

### 4.1 Liver cancer

**FIGURE 4:**
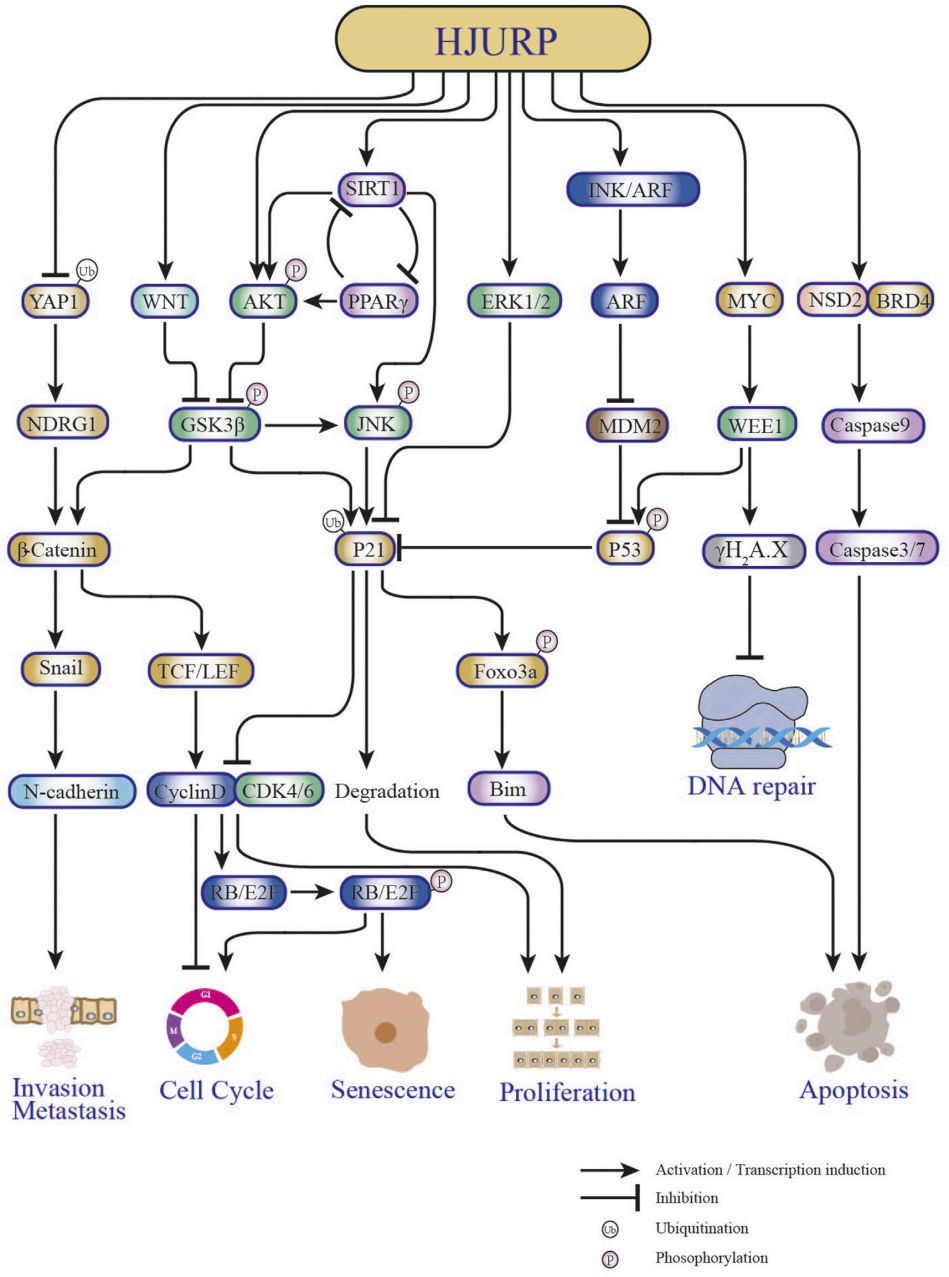
Key pathways in tumorigenesis and progression affected by HJURP.

Several studies have shown that HJURP expression is significantly upregulated in hepatocellular carcinoma (HCC) tissues compared to paraneoplastic tissues (Lan, 2016; Du, 2017; Chen et al., 2018; Chen T. et al., 2019) (Table 1). There was also a significant correlation between upregulated HJURP expression and clinical features such as tumor lesion >5 cm (Lan, 2016; Hu et al., 2017), tumor stage (AJCC) (Chen et al., 2018), BCLC stage, and microvascular invasion (Hu et al., 2017). It was further suggested that high expression of HJURP may predict lower disease-free survival and higher likelihood of microvascular infiltration, serving as an independent factor for poor prognosis in HCC patients (Hu et al., 2017; Chen T. et al., 2019). In another study, Chen et al. found that high HJURP expression was associated with low p21 expression and unsatisfactory clinical outcomes (Chen et al., 2018). In addition, functional experimental studies showed that HJURP acts as a tumor driver, promoting proliferation, migration, invasion, and epithelial-mesenchymal transition (EMT) and inhibiting apoptosis in HCC cells (Yi, 2015; Hu et al., 2017; Chen et al., 2018; Chen T. et al., 2019). Specifically, it was reported that HJURP promotes HCC cell survival by regulating the nuclear-cytoplasmic translocation and ubiquitin-mediated degradation of p21 through the MAPK/ERK1/2 and AKT/GSK3β pathways, thus promoting G1/S phase transition (Chen et al., 2018). On the other hand, HJURP activates EMT through upregulation of Sphingosine kinase 1 (SPHK1), causing upregulation of vimentin and N-cadherin and downregulation of E-cadherin to promote tumor cell migration, invasion, and metastasis *in vivo* (Chen T. et al., 2019) (Figure 4). In a recent study employing bioinformatics analysis and immunohistochemistry, Yang et al. reported that HJURP was highly expressed in cholangiocarcinoma (CCA) samples (Yang et al., 2022). High expression of HJURP was associated with low overall survival in intrahepatic CCA and perihilar CCA, but not in distal CCA (Yang et al., 2022). Luo et al. reported that high HJURP expression in HCC was associated with tumor-infiltrating immune cells, immune checkpoints, and immunosuppression, verified by gene sequencing analysis (Luo et al., 2022). Their study further proposed a prognostic risk score model based on HJURP-related genes predicted to be involved in immune responses within the tumor microenvironment. Although more detailed studies on the mechanisms by which HJURP promotes HCC and CCA, as well as its impact on tumor immunogenicity, are warranted, these findings suggest that HJURP is a potential biomarker and therapeutic target for HCC and CCA.

**TABLE 1:** The role and underlying mechanisms of HJURP in various cancers.

Types	Expression	Role	Associated with clinical characters	Prognosis	Function	Related genes/pathway	Refs
Liver cancer	Upregulated	Oncogene	Tumor size, Tumor stage (AJCC)	Poor	Proliferation, cell cycle	CDKN1A MAPK/ERK1/2 AKT/GSK3β	Chen et al. (2018)
	Upregulated	Oncogene	Microvascular invasion	Poor	Migration, invasion, and EMT	SPHK1	Chen et al. (2019a)
	/	/	/	/	Proliferation, migration, invasion, apoptosis, and cell cycle	/	Yi (2015)
	Upregulated	/	Tumor stage, Child-Pugh	Poor	/	/	Du (2017)
	Upregulated	/	Tumor size, AFP, Vessel carcinoma embolus	/	/	/	Lan (2016)
	Upregulated	/	Tumor size, BCLC stage	Poor	Proliferation	/	Hu et al. (2017)
Renal cell carcinoma	Upregulated	Oncogene	Tumor stage, clinical Staging classification, Histopathological stage, immunocyte infiltration	Poor	/	/	Zhang et al. (2021)
	Downregulation	Tumor suppressor genes	/	/	Proliferation, apoptosis, cell cycle, and oxidative stress	CDKN1A PPARγ/SIRT1 FOXO3a	Yuan et al. (2020a)
	Upregulated	Oncogene	/	Poor	Proliferation, migration	AKT	Patel et al. (2014)
	Downregulation	Tumor suppressor genes	/	/	/	/	Yuan et al. (2020b)
Breast cancer	Upregulated	Oncogene	Tumor size	Poor	Proliferation	YAP1/NDRG1	Mao et al. (2022)
	Upregulated	Oncogene	/	Poor	/	/	Montes de Oca et al. (2015)
	Upregulated	Oncogene	Estrogen receptor (ER), progesterone receptor (PR), Scarff-Bloom-Richardson (SBR) grade, age, and Ki67 proliferation indices	Poor	Proliferation	/	Hu et al. (2010)
Prostate cancer	Upregulated	Oncogene	/	Poor	Proliferation, cell cycle	CDKN1A GSK3β/JNK	Lai et al. (2021)
	Upregulated	Oncogene	Gleason grade, Pathological stage	Poor	Proliferation, metastasis	/	Chen et al. (2019b)
Glioma	Upregulated	Oncogene	/	/	Proliferation, cell cycle, and apoptosis	/	Serafim et al. (2020)
	Upregulated	Oncogene	/	Poor	Proliferation, cell cycle, and apoptosis	/	Valente et al. (2013)
	Upregulated	Oncogene	Tumour grade	Poor	invasion	/	De Tayrac et al. (2013)
	Upregulated	Oncogene		Poor	Proliferation, migration	KLF11	Li et al. (2022)
Gastric carcinoma	Upregulated	Oncogene	Lymphatic metastasis, TNM Stage, vascular cancer plug	Poor	/	/	Wang et al. (2021)
	Upregulated	Oncogene	TNM Stage	Poor	/	/	Chi (2017)
Lung cancer	Upregulated	Oncogene	TNM stage	Poor	Proliferation, migration, and invasion	Wnt/β-catenin	Wei et al. (2019)
	Upregulated	Oncogene	/	Poor	/	/	Chen and Wang (2021)
Bladder cancer	Upregulated	Oncogene	/	/	Proliferation, apoptosis, and cell cycle	PPARγ-SIRT1	Cao et al. (2017)
Oral cancer	Upregulated	Oncogene	/	Poor	Proliferation, cell cycle	/	Tsevegjav et al. (2022)
Multiple myeloma	Upregulated	Oncogene	/	Poor	Proliferation, apoptosis	NSD2/BRD4	Jia et al. (2022)
Ovarian serous cystadenocarcinoma	Upregulated	Oncogene	/	Poor	Proliferation, migration, invasion, and cell cycle	MYC/WEE1	Dou et al. (2022)
Pancreatic cancer	Upregulated	Oncogene	/	Poor	Proliferation, migration, and invasion	MDM2/p53	Wang et al. (2020a)

### 4.2 Renal cell carcinoma

Analysis of tumor mRNA expression profiles in the TCGA database revealed that the expression of HJURP was higher in kidney cancer than in normal adjacent tissues (Zhang et al., 2021). Overexpression of HJURP and clinicopathological features of kidney cancer patients, such as T-stage, N-stage, M-stage, clinical stage, and pathological grade, were significantly correlated (Table 1). Interestingly, HJURP expression was strongly and positively correlated with immune cell infiltration, and associated with marker genes of Tregs and exhausted T-cell such as FOXP3, TGB1, PD-1, CTLA4, LAG3, TIM-3, and GZMB. Furthermore, survival analysis showed that overall survival was lower in renal cancer patients with high HJURP expression (Zhang et al., 2021). A previous study had shown that HJURP silencing inhibited the proliferation of human renal cancer CAKI-1 cells, arresting them in G2/M phase, and diminished their migratory capacity by inhibiting EMT and downregulating p-AKT and p-GSK3β (Patel et al., 2014). This evidence suggested that HJURP may promote the invasive migration of kidney cancer cells through the AKT signaling pathway. While these studies suggested that HJURP plays a pro-oncogenic role, HJURP may also exert antitumoral properties in renal cancer. When overexpressed, HJURP was found to inhibit the proliferation of A498 kidney cancer cells, induce an increase in apoptosis, and block the cell cycle at the G0/G1 phase (Yuan et al., 2020a). It was further shown that HJURP induces apoptosis in kidney cancer cells by promoting ROS accumulation through inactivation of PPARγ/SIRT1 and downstream Forkhead box O3 (FOXO3a) signaling (Yuan et al., 2020a) (Figure 4). Therefore, whether HJURP plays a pro- or anti-cancer role in renal cell carcinoma deserves further investigation. In addition, there is evidence that HJURP promotes immune escape in renal cancer (Zhang et al., 2021), an interesting phenomenon whose driving mechanism deserves also further enquiry.

### 4.3 Breast cancer

Analysis of a large panel of breast cancer (BC) cell lines revealed upregulated levels of HJURP expression in approximately 50% of cell strains, compared to immortalized non-malignant breast epithelial cells. Moreover, HJURP mRNA levels were significantly higher in invasive ductal carcinoma than in normal breast duct cells (Hu et al., 2010). HJURP mRNA levels were significantly correlated with estrogen receptor (ER), progesterone receptor (PR), Scarff-Bloom-Richardson (SBR) grading, age, and Ki67 proliferation index, but not with pathological stage, ERBB2, tumor size, or lymph node status, and showed an association with shortened disease-free survival and overall survival in BC patients (Hu et al., 2010) (Table 1). These findings led to propose HJURP as an independent prognostic factor for BC, with a better predictive value than Ki67, able also to aid the prognosis of different molecular subtypes (i.e., normal-like, luminal, Erbb2, and basal) (Hu et al., 2010). Bravaccini et al. found that patients with *in situ* BC presenting high HJURP expression in the mesenchyme had a greater than 7-fold higher risk of recurrence, and suggested that HJURP could be used as a marker for the biological evolution of *in situ* BC (Bravaccini et al., 2014). Interestingly, Hu et al. concluded that BC patients with high HJURP expression respond better to radiotherapy, and that HJURP can be used as a marker of radiotherapy sensitivity (Hu et al., 2010). Accordingly, HJURP knockdown in human BC cells reduced their sensitivity to radiation treatment (Hu et al., 2010; Bravaccini et al., 2014). Recently, Mao et al. reported for the first time the potential regulatory mechanism of HJURP in triple-negative BC (TNBC), which typically presents higher expression of HJURP compared to other subtypes (Mao et al., 2022). HJURP is reported to have the function of regulating protein ubiquitination (Chen et al., 2018; Lai et al., 2021). Interestingly, they found that HJURP can bind to Yes associated protein 1 (YAP1) protein and then reflect its ubiquitination by lysosome-dependent degradation in TNBC (Mao et al., 2022). In addition, it was demonstrated that overexpression of YAP1 rescued both inhibition of cell growth and adriamycin sensitivity after HJURP knockdown (Mao et al., 2022). N-Myc downstream regulated gene 1 (NDRG1) is involved in the apoptotic process in a variety of tumors (Yan et al., 2008; Jung et al., 2010; Ito et al., 2020). Mao et al. further demonstrated that the activation of the HJURP/YAP1 regulatory axis increases the transcriptional level of NDRG1, implicating the HJURP/YAP1/NDRG1 pathway in the proliferation and doxorubicin resistance of TNBC (Mao et al., 2022) (Figure 4). Although HJURP has arisen as a potential biomarker for BC and a promising predictive indicator of sensitivity to tumor radiotherapy, further evaluation and validation of HJURP as a predictive marker for the diagnosis and prognosis of multimodal treatment of BC is clearly needed (Coates et al., 2010).

### 4.4 Prostate cancer

A recent study by Lai et al. showed that compared to human normal prostate epithelial cells, HJURP protein levels were significantly higher in prostate cancer (PCa) cell lines (Lai et al., 2021). Moreover, the expression of HJURP was also upregulated in PCa compared with benign prostatic hyperplasia tissues, and correlated positively with Gleason grade (Lai et al., 2021). Previously, based on analysis of an online PCa dataset (Taylor dataset), Chen et al. had shown that HJURP upregulation was significantly associated with PSA levels, high Gleason grade, disease progression, metastasis, and PSA failure (Chen Y. F. et al., 2019). In addition, Zhou et al. demonstrated a significant association between elevated HJURP and shorter biochemical relapse (BCR) progression-free survival (PFS) in PCa (Zhou et al., 2017) (Table 1). Consistently, HJURP protein expression was shown to be an independent predictor of poor overall survival in PCa patients by univariate and multifactorial analyses (Lai et al., 2021). The latter study also showed that HJURP knockdown inhibits the proliferation of PCa cells *in vivo* and *in vitro*; conversely, overexpression of HJURP promotes tumor proliferation (Lai et al., 2021). Mechanistically, HJURP regulates p21 ubiquitination through the GSK3β/JNK signaling pathway and promotes p21 ubiquitin-dependent proteasomal degradation; this in turn promotes G1/S phase transition, ultimately leading to PCa progression (Lai et al., 2021) (Figure 4). Although further studies are needed, the above evidence suggests that HJURP may be a useful prognosis marker and a new target for PCa treatment.

### 4.5 Glioma

HJURP is highly expressed in low-grade diffuse (grade II) astrocytoma, anaplastic (grade III) astrocytoma, and glioblastoma (grade IV) (Valente et al., 2009; De Tayrac et al., 2013). A meta-analysis incorporating four public repositories and datasets (REMBRANDT, TCGA, GSE4271, and GSE4412) showed an association between high HJURP expression and poorer survival prognosis in glioma patients (De Tayrac et al., 2013) (Table 1). Combining other genes such as EDN/RB and p60/CAF-1 had better predictive power for malignant glioma (De Tayrac et al., 2013). Silencing of HJURP in glioma cells resulted in prominent morphological changes paralleled by significantly decreased viability, increased apoptosis, cell cycle arrest in the G2/M phase, and accelerated senescence (Serafim et al., 2020). Furthermore, HJURP knockdown resulted in a severe reduction in CENP-A levels and no detectable aggregation of CENP-A on centromeres. Thus, the impact of HJURP on glioma progression may be related to the loss of CENP-A from the centromere, with subsequent deficiency in the maintenance of post-mitotic centromeric structure (Serafim et al., 2020). Interestingly, and contrasting with the effect observed in BC cells, HJURP silencing increased radiotherapy-induced death in glioma cell lines. In turn, Li et al. demonstrated that either HJURP or Kruppel-like factor 11 (KLF11) silencing inhibited glioma cell proliferation and migration, while HJURP overexpression partially rescued these effects in cells deficient in KLF11 (Li et al., 2022). It was thus concluded that KLF11 promotes the progression of glioma through positive regulation of HJURP, and that both proteins could be used as biomarkers for glioma diagnosis and prognosis (Li et al., 2022). However, further studies are needed to determine the value of these findings for clinical and therapeutic management.

### 4.6 Lung cancer

HJURP was shown to be highly expressed in 23 non-small cell lung cancer (NSCLC) cell lines, but not expressed in small airway epithelial cells derived from normal bronchial epithelial cells (Kato et al., 2007). Similarly, significantly higher HJURP expression was detected in clinical NSCLC samples compared to paraneoplastic tissues. HJURP expression in NSCLC was also significantly correlated with clinicopathological features such as pathological stage, TNM stage, and distal metastasis (Kato et al., 2007) (Table 1). Wang et al. applied a comprehensive bioinformatics approach and found that HJURP is a hub gene in NSCLC, associated with poor prognosis (Wang L. et al., 2020). Fu et al. constructed a 5-gene model, including HJURP, TYMS, CDK1, CEP55, and KIF11, to predict brain metastasis after lung cancer surgery and validated it in two independent datasets (Fu et al., 2020). Interestingly, Zhou et al. found that HJURP mRNA expression in plasma could be used as a novel non-invasive biomarker for lung cancer (Zhou et al., 2017). The study, which included 47 lung cancer patients and 14 healthy subjects, showed that the expression of HJURP was significantly upregulated in lung cancer patients. At the optimal cut-off point, plasma HJURP separated lung cancer patients from healthy individuals with a sensitivity of 66.0%, a specificity of 78.6%, and an AUC (area under the ROC) of 0.696 (Zhou et al., 2017). Meanwhile, Wei et al. showed that functional silencing of HJURP inhibited the proliferation of NSCLC cells, accelerated apoptosis, and suppressed their invasion and migration capacities (Wei et al., 2019). Mechanistically, knockdown of HJURP in NSCLC cells decreased the expression of β-catenin, cyclin D1, and c-myc, suggesting that HJURP may promote tumor progression by stimulating the Wnt/β-catenin pathway (Wei et al., 2019). In a recent study, Chen et al. analyzed HJURP mRNA expression and associated clinical parameters in 480 lung adenocarcinoma (LUAD) patients from the TCGA database, and suggested that high HJURP expression may be an independent prognostic factor and a potentially useful prognostic molecular biomarker for low survival in LUAD cases. In addition, such study showed that HJURP expression correlated with immune cell infiltration (Chen et al., 2022). The above evidence thus suggests that HJURP represents a promising diagnostic and prognostic marker for lung cancer. Still, whether detection of HJURP in plasma might aid the diagnosis of lung cancer, and the potential involvement of HJURP in tumor-associated immune infiltration, are topics worth of further investigation.

### 4.7 Ovarian serous cystadenocarcinoma

A recent study revealed that mRNA and protein levels of HJURP in Ovarian serous cystadenocarcinoma (OV) tissues were significantly higher than those detected in normal fallopian tube; consistently, HJURP was also overexpressed in OV cell lines compared to normal ovarian epithelial cells (Dou et al., 2022). High expression of HJURP was shown to be a negative risk factor for PFS by univariate and multifactorial Cox regression analyses, whereas Kaplan-Meier analysis indicated an association between high HJURP expression and worse prognosis, evaluated by both OS and PFS, in OV patients (Dou et al., 2022). This is consistent with the previous findings by Li et al., who showed that increased expression of HJURP is an independent negative prognostic biomarker in patients with advanced serous OV (Li et al., 2018) (Table 1). Silencing of HJURP significantly inhibited OV cell proliferation by inducing G0/G1 arrest and decreased also the migration and invasion potential of these cells. Of note, although HJURP silencing in OV cells did not promote apoptosis, this process was enhanced when combined with cisplatin treatment (Dou et al., 2022). Interestingly, a positive association between HJURP expression and EMT was established by assays that showed that both vimentin and Slug expression was downregulated after HJURP knockdown, whereas Slug expression was significantly upregulated upon HJURP overexpression in SKOV3 cells (Dou et al., 2022). This finding is thus consistent with the study by Chen et al., which showed that HJURP promotes EMT in HCC (Chen T. et al., 2019). Based on gene enrichment analysis, cell cycle, DNA replication, and DNA integrity checkpoints were identified by Dou et al. as major functional processes impacted by HJURP in OV. In addition, the study indicated that the G2/M checkpoint kinase WEE1 is a downstream target of HJURP (Dou et al., 2022). Further analysis confirmed that HJURP regulates WEE1 through the transcription factor MYC and promotes cisplatin chemotherapy resistance through the MYC/WEE1 axis (Dou et al., 2022). Furthermore, silencing of HJURP increased sensitivity to AZD1775, a small molecule inhibitor of WEE1, and promoted DNA repair after cisplatin-induced DNA damage (Dou et al., 2022) (Figure 4). Interestingly, another recent study showed that HJURP silencing reduced mitochondrial contents in OV cells, thereby inhibiting proliferation and promoting apoptosis. It was further determined that HJURP inhibits the proliferation of OV cells by regulating CENP-A/Centromere Protein N (CENP-N) (Zhang et al., 2022). Thus, the above evidence indicates that HJURP is highly expressed and has an important prognostic value in OV, providing also an important avenue to treat cancer through a mitochondria-targeted approach. Although the potential impact of HJURP on radiotherapy sensitivity in OV has not yet been studied, future studies may provide a theoretical basis for treatments combining HJURP targeting, radiotherapy, and chemotherapy.

### 4.8 Pancreatic cancer

Wang et al. reported that HJURP is highly expressed in clinical pancreatic ductal adenocarcinoma (PDAC) tissues, and that patients with high tumoral HJURP levels have significantly poorer survival compared to patients with low HJURP (Table 1). In turn, overexpression and knockdown experiments in PDAC cell lines, as well as analysis of mouse xenograft models, showed that HJURP promoted viability, tumorsphere formation, and migration and invasion *in vitro*, and tumor growth and metastasis *in vivo* (Wang C. J. et al., 2020). The study found that HJURP regulates Murine double minute2 (MDM2) expression through H3K4me2, and that the MDM2/p53 axis is critically involved in HJURP-mediated malignant behavior in PDAC (Wang C. J. et al., 2020) (Figure 4). Thus, although research on the role of HJURP in pancreatic cancer is still limited, the above evidence suggests the potential value of HJURP as a biomarker and target for the prognosis and treatment of PDAC.

### 4.9 Multiple myeloma

Jia et al. showed that transcript and protein expression levels of HJURP were upregulated in myeloma cells carrying the t (4; 14) translocation and that HJURP overexpression was associated with poor clinical survival (Jia et al., 2022) (Table 1). Cell experiments showed also that overexpression of HJURP promoted cell growth, suppressed apoptosis, and was functionally relevant in both t (4; 14) and non-t (4; 14) myeloma (Jia et al., 2022). The study also revealed that transcriptional activation of the HJURP gene in t (4; 14) myeloma resulted from the presence of a super-enhancer (SE) distal to the HJURP gene promoter, and was induced by binding of NSD2/BRD4 complexes to those two regions (Jia et al., 2022) (Figure 4). These results suggested that HJURP is involved in carcinogenesis and subsequent progression of multiple myeloma, and may represent a novel therapeutic target for t (4; 14)-positive patients.

### 4.10 Bladder cancer

A study by Cao et al. showed that HJURP expression was markedly upregulated in bladder cancer (BCa) tissues compared to both adjacent non-cancerous tissues and normal bladder tissues (Cao et al., 2017). HJURP silencing inhibited significantly the proliferation of BCa cells, but migration was not affected. In turn, flow cytometry analysis showed that the number of early apoptotic cells was increased after HJURP silencing and that the latter induced cell cycle arrest at the G0/G1 phase (Table 1). Moreover, several proteins involved in ROS metabolism (catalase, Hmox-1, SOD2) were significantly upregulated after HJURP knockdown. In addition, HJURP silencing increased PPARγ and suppressed p-SIRT1/t-SIRT1 protein expression. These findings therefore indicated that HJURP regulates ROS metabolism, apoptosis, and cell cycle in BCa cells. Interestingly, protein expression levels of acetylated p53 were increased in HJURP knockdown cells, especially in the nuclear region, a phenomenon controlled by the negative feedback of SIRT1 deacetylase, linking metabolism and apoptosis. It was also revealed that CENP-A was positively correlated with HJURP and involved in the regulation of cell cycle in BCa cells (Cao et al., 2017). In summary, the above study showed that HJURP is highly expressed in BCa, promoting its progression *via* regulation of ROS metabolism and cell cycle through a PPARγ-SIRT1 feedback loop (Figure 4). However, the presence of a direct regulatory link between HJURP and the PPARγ-SIRT1 axis in bladder carcinogenesis needs to be further ascertained.

### 4.11 Oral cancer

Tsevegjav et al. showed that HJURP was highly expressed, at both mRNA and protein levels, in oral cancer (OC) cells and tissues (Tsevegjav et al., 2022). In turn, Kaplan-Meier analysis showed that HJURP protein expression was positively associated with worse prognosis in OC patients, while multivariate analysis indicated that positive HJURP expression and advanced pN stage were independent prognostic factors (Table 1). HJURP silencing led to cell cycle arrest and induced cellular senescence, whereas inhibition of the HJURP-CENP-A interaction significantly inhibited the growth of OC cells (Tsevegjav et al., 2022). In addition, tissue microarray analysis showed that HJURP expression was not associated with the size of OC tumors. Therefore, targeting HJURP expression and/or its interaction with CENP A may be a valid therapeutic goal in the development of new drugs for OC. However, since the exact molecular mechanism of HJURP activation in OC and its oncogenic effects have not been fully elucidated, further detailed analysis is warranted (Tsevegjav et al., 2022).

### 4.12 Other tumors

HJURP is also overexpressed and plays a tumor-promoting role in gastric carcinoma (GC) (Wang et al., 2021)and colorectal cancer (CRC) (Kang et al., 2020). Wang et al. found that HJURP expression correlated significantly with lymphatic metastasis, TNM stage, and the presence or absence of cancerous vascular thrombi in GC tissue samples from an internal cohort (Wang et al., 2021). Meanwhile, Kang et al. observed that HJURP is moderately or highly expressed in CRC tissues and weakly or not expressed in normal colon epithelial cells (Kang et al., 2020). Their study detected an inverse association between HJURP expression and CRC prognosis, determined that silencing of HJURP inhibited the proliferation, migration, invasion, and tumorigenicity of CRC cells, and suggested that HJURP might be a potential prognostic biomarker and a new target for drug discovery in CRC (Kang et al., 2020). However, as the mechanisms underlying HJURP-induced malignant behavior in CRC have not yet been elucidated, further studies on the oncogenic role of HJURP in CRC would be of great significance. In summary, HJURP has significant oncogenic effects in multiple tumor types. Further studies on its expression patterns, function, and molecular interactions in other cancer types should provide additional meaningful insights into the ubiquitous role of HJURP in cancer progression and help confirm its prognostic and therapeutic significance.

## 5 Conclusion and future perspectives

HJURP functions as a specific CENP-A chaperone, supporting the localization of CENP-A on centromeric chromatin and assisting in centromeric assembly. Numerous studies suggested that HJURP plays a pro-oncogenic role in different types of cancer. HJURP is overexpressed in a variety of cancers, including liver, renal cell, prostate, glioma, breast, stomach, lung, bladder, oral cavity, multiple myeloma, colorectal, and pancreatic cancer. Collectively, the evidence indicates that HJURP overexpression is substantially associated with poor patient prognosis. Knockdown of HJURP inhibits proliferation, migration, invasion, colony formation, and EMT, and may promote apoptosis in different tumor cells. Although this evidence indicates that HJURP has great potential in the diagnosis, prognosis, and treatment of cancer, further research on the regulatory mechanisms of HJURP in different neoplasia is necessary. Below, we propose some ideas that we hope will serve as directions for future in-depth explorations. First, the role of HJURP in cancer has so far been addressed in a limited range of tumor types; thus, further experiments are needed to confirm whether HJURP is involved in the occurrence and development of other cancers. Second, among existing studies, robust evidence of an association between HJURP and cancer progression *in vitro* has been gathered, but to date few experimental data, limited mostly to xenograft and lung metastasis models, provided insight into the pro-oncogenic role of HJURP *in vivo*. Third, tumor chemo- and radio-resistance are still main obstacles to improving the prognosis of cancer patients; further research on the modulation of HJURP in tumor cells may prove very valuable for improving drug sensitivity and reducing radiotherapy resistance. Fourth, peripheral blood is a very valuable diagnostic medium, yet the diagnostic potential of non-invasive screening of circulating HJURP in cancer patients has received little attention. Lastly, although it has been verified that HJURP can promote the occurrence and development of tumors, the impact of HJURP on the tumor microenvironment has been insufficiently studied and requires therefore further research.

In summary, although HJURP was shown to play an important role in tumorigenesis and development, the molecular mechanisms involved are complex and remain incompletely characterized. Therefore, further researches are warranted to confirm the value of HJURP as a diagnostic, prognostic and therapeutic target in cancer.
